# The relationship between vitamin D levels and depression: a genetically informed study

**DOI:** 10.1186/s12937-025-01199-1

**Published:** 2025-10-10

**Authors:** Honggang Lyu, Lijun Kang, Qian Gong, Xin-Hui Xie, Simeng Ma, Lihua Yao, Mian-mian Chen, Lingfeng Zhang, Hao Yu, Xubo Wang, Chao Wang, Zhongchun Liu

**Affiliations:** 1 Department of Psychiatry, Renmin Hospital of Wuhan University, 238 Jiefang Road, Wuhan, 430060 China; 2 Department of Psychiatry, Jining Medical University, Jining, Shandong China; 3 Department of Psychiatry, Shandong Daizhuang Hospital, Jining, Shandong China; 4 Taikang Center for Life and Medical Sciences, Wuhan University, Wuhan, 430071 China

**Keywords:** Vitamin d levels, Depression, Genetic overlap, Conjunctional false discovery rate (conjFDR), Genetic causality

## Abstract

**Background:**

Low vitamin D (vitD) levels are consistently associated with an increased risk of depression. However, the biological mechanisms underlying this relationship and potential shared genetic overlap remain elusive.

**Methods:**

We investigated the genetic overlap and causal relationships between depression (*N* = 589,356) and vitD levels (*N* = 417,580) using genome-wide association study (GWAS) summary statistics. We performed genome-wide and local genetic correlation analyses, followed by quantification of polygenic overlap variants. Shared genetic loci were identified and mapped to genes, which were further analyzed through gene expression and lifespan brain expression trajectory analyses. Bidirectional causal relationships were examined using multiple Mendelian randomization approaches.

**Results:**

We observed significant negative genetic correlations (*r*_*g*_ = -0.079) and identified genetic overlap (*N* = 410 variants). Genes mapped to the 13 shared loci showed opposing expression patterns. Tissue- and cell-specific functional enrichment analyses revealed significant signals related to brain development, with distinct patterns emerging between fetal development and adulthood. Shared genes (*TRMT61A*, *ITIH4*, *RASGRP1*, *CTNND1*, *HERC1*, *IP6K1*, *FURIN ESR1*, *ZMYND* and *GRM5*) exhibited notable expression variation in the brian throughout the lifespan, aligning with functional enrichment findings.

**Conclusions:**

Our findings elucidate the shared biological mechanisms underlying the relationship between vitD and depression, suggesting that vitD play an important role in the development of depression through altered early neurodevelopmental processes.

**Supplementary Information:**

The online version contains supplementary material available at 10.1186/s12937-025-01199-1.

## Background

Depression is a major global mental health challenge and the leading cause of years lived with disability (YLDs) among all non-communicable diseases. It is a common and frequently recurrent condition, with a global prevalence of 4.4%, affecting an estimated 332 million people worldwide in 2021 [1, 2]. Depression affects people across the lifespan, particularly in the 15–19 and 60–64 age groups [3], imposing a substantial burden on both public health systems and individuals. Although numerous hypotheses have been proposed over the past centuries, such as neuroinflammation, monoamine dysfunction, genetic factors, and functional brain abnormalities, the pathophysiology of depression remains elusive [1]. Consequently, identifying modifiable factors for depression could inform effective prevention strategies.

The role of vitamin D (vitD) has received significant attention in research investigating its potential for the prevention and treatment of depression [4]. An increasing number of preclinical and clinical studies suggests that vitD deficiency may increase depression risk, while supplementation is associated with reduced depressive symptom severity scores [4]. As a fat-soluble vitamin, vitD plays a critical role in regulating calcium and phosphate levels in the body and participates in various physiological mechanisms. With emerging insights into its functions, vitD has garnered considerable attention for its anti-inflammatory, pro-neurogenic, and neuromodulatory properties, which have been shown to play a vital role in its antidepressant effects [5–7]. Several systematic reviews and meta-analyses have reported that individuals with the lowest vitD levels exhibit significantly higher depression risk compared to those with the highest levels for various populations including postpartum [8], older adults [9] and adults in general [10]. Correspondingly, analyses of vitD supplementation have demonstrated a beneficial impact on depressive symptom severity [11], as well as the incidence and prognosis of depression [12]. Multiple factors may be involved in the neurobiological mechanisms underlying the comorbidity of the two conditions. Neurons and glial cells express vitD receptors in brain regions such as the prefrontal cortex and hippocampus, suggesting a possible role for vitD in mood regulation and its potential influence on the progression of depression [13]. Vitamin D deficiency (VDD) during pregnancy has been shown to impact brain development in offspring, leading to a thinner cortex and reduced expression of genes and proteins associated with synaptic plasticity, neurotransmission, and levels of nerve growth factor [14–16]. However, both traits are complex conditions influenced by multiple factors, and the common underlying biological mechanisms remain largely unclear. The shared genetic foundation may illuminate the relationship between vitD and depression, providing insights into shared biological mechanisms.

Genome-wide association studies (GWAS) have advanced our understanding of the biological mechanisms underlying complex traits [17]. Recent large-scale GWAS on depression identified 246 genetic risk loci associated with genes involved in brain development, signal transduction, inflammation, and metabolism [18]. Similarly, GWAS of vitD identified 143 loci associated with vitD levels, implicating genes related to lipid metabolism [19]. A significant negative genetic correlation between vitD and depression was evaluated using linkage disequilibrium score regression (LDSC) [19]. However, LDSC may not fully capture genetic overlap with mixed-effect directions, a common feature of complex phenotypes [20, 21]. The bivariate causal mixture model (MiXeR), developed by Frei et al., addresses this limitation by providing a more robust framework for assessing genetic overlap irrespective of effect direction, thus enabling quantification of shared polygenic architecture [22–24]. Given that both phenotypes are associated with numerous small-effect genetic variants, cross-trait meta-analysis presents an effective strategy for detecting shared genetic signals and identifying common genetic loci [25]. In this context, Jaholkowski et al. investigated the shared genetic architecture between schizophrenia and vitD levels, revealing novel risk loci that illuminate overlapping biological mechanisms [26]. However, to our knowledge, the shared genetic basis between depression and vitD levels remains unexplored using large-scale genomic datasets.

**Fig. 1:**
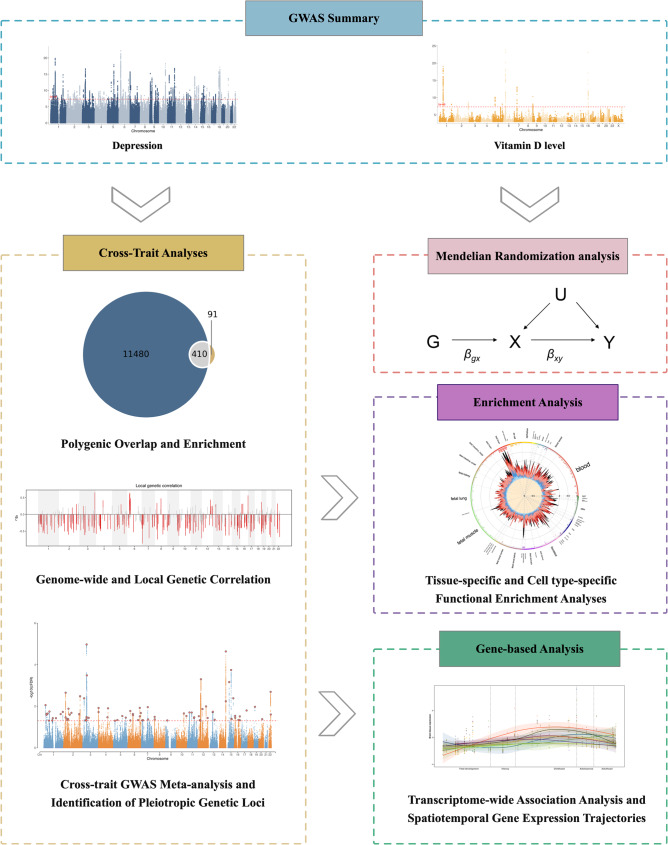
Schematic workflow of the study design

In this study, we examined the genetic basis of epidemiological associations between vitD levels and depression using large-scale genomic datasets. We first assessed genetic correlations and quantified variants with polygenic overlap. Through Multi-Trait Analysis of GWAS (MTAG), we conducted cross-trait meta-analysis to identify variants jointly associated with both phenotypes. To validate our findings and address potential MTAG assumption violations, we performed a complementary conjunctional false discovery rate (conjFDR) analysis. Gene-based analyses using transcriptome-wide association studies (TWAS) integrated data from Spatiotemporal Transcriptome of the Human Brain, GTEx, and PsychENCODE, to identify shared gene expression patterns. This comprehensive genetic investigation elucidates the biological mechanisms underlying the relationship between depression and vitD levels, potentially informing preventive strategies and identifying novel therapeutic targets. The study design is presented schematically in Fig. 1.

## Methods and materials

### GWAS summary statistics

In this current study, we accessed GWAS summary statistics for depression from the iPSYCH consortium (https://ipsych.dk/), representing the largest dataset to date, which includes 22 cohort studies with 102,084 depression cases and 771,257 controls [18]. Following the exclusion of 23andMe samples, our analysis included iPSYCH GWAS summary data from 589,356 individuals (48,975 cases and 540,381 controls). For serum vitD levels, we obtained GWAS summary statistics from the Program in Complex Trait Genomics (https://cnsgenomics.com/content/research), involving data from 417,580 individuals [19]. The majority of participants across all studies were of European ancestry. Detailed information on GWAS quality control and association analyses can be found in the original studies.

### Genetic overlap and correlations

We employed Bivariate MiXeR to examine the genetic overlap between vitD and depression, quantifying shared trait-influencing variants between the two phenotypes [22]. MiXeR calculates both the proportion of shared variants relative to the total number of variants influencing both phenotypes and the fraction of these shared variants with concordant effects. The estimated quantities of shared and trait-specific variants were illustrated using Venn diagrams. Model adequacy was evaluated using the Akaike Information Criterion (AIC), where a positive value indicates sufficient model power and supports the reliability of MiXeR estimates. Additionally, we constructed conditional Quantile–Quantile (Q-Q) plots to depict cross-trait polygenic enrichment [27]. Enrichment for shared genetic effects is demonstrated in conditional Q-Q plots as successive leftward deflections from the null distribution. We evaluated genetic correlations at both genome-wide and local regional levels. At the genome-wide level, we employed linkage disequilibrium score regression (LDSC) and high-definition likelihood (HDL) to assess genetic correlation [21, 28, 29]. LDSC, the most commonly used primary approach, leverages pre-calculated linkage disequilibrium (LD) scores from the European reference population of the 1,000 Genomes Project. HDL extends the LDSC regression framework by implementing a full likelihood model and incorporating complete LD score matrix information [29]. HDL analysis was conducted using pre-calculated LD scores from a reference panel of 336,000 British genomic samples from the UKBiobank. Moreover, both LDSC and HDL are robust to potential sample overlap. At the local regional level, we applied the local analysis of co-variant annotation (LAVA) to examine local SNP heritabilities and genetic correlations across 2,495 predefined genomic regions [30]. We first conducted univariate LAVA analysis to quantify local heritability for each phenotype. Regions showing significant heritability after Bonferroni correction (*p* < 0.05/2,495) were selected for local genetic correlation (*r*_*g*_) analysis. For genomic loci exhibiting significant heritability in both vitD and depression, bivariate LAVA analysis was conducted to calculate the local genetic correlation. The results were visualized using Manhattan-style plots.

### Cross-trait GWAS meta-analysis and identification of shared genetic loci

To explore the shared genetic etiology between vitD levels and depression, we performed cross-trait GWAS meta-analysis using multitrait analysis of GWAS (MTAG) [25]. MTAG applies generalized inverse-variance-weighted meta-analysis to multiple traits while accounting for sample overlap between GWAS datasets, enhancing statistical power for identifying pleiotropic loci [25]. Given that MTAG assumptions, such as equal single-nucleotide variants (SNVs) heritability for each trait and identical genetic covariance between traits, may be violated, we conducted a complementary conjunctional false discovery rate (conjFDR) analysis to identify pleiotropic genetic loci [27, 31]. The conjFDR method employs an empirical bayesian framework that leverages cross-trait SNP enrichment to enhance detection of variants jointly associated with both phenotypes [23, 24, 32]. A conjFDR threshold of < 0.05 was used to identify shared genetic loci between vitD and depression.

For pleiotropic genetic loci identified through both two approaches, we assessed the directional effects of shared loci by comparing their GWAS summary Z-scores. We employed the FUMA protocol (http://fuma.ctglab.nl/) to identify the SNVs with the most significant p-value as the lead SNP for each genetic locus [33]. The shared lead SNVs were mapped to their nearest genes based on physical proximity and functional consequences using three approaches: Combined Annotation Dependent Depletion (CADD) scores [34], RegulomeDB scores [35], and chromatin state annotations via FUMA. Additionally, positional mapping was performed to assign lead SNVs to their nearest genes. Loci that did not physically overlap with findings from the original GWASs [18, 19], and the GWAS Catalog [36] were considered novel.

### Tissue-specific and cell-type-specific functional enrichment analysis

We performed tissue-specific and cell-type-specific enrichment analyses using GWAS summary statistics from our MTAG meta-analysis to characterize the functional significance of shared genetic signals. For tissue-specific, we utilized the Multi-marker Analysis of Genomic Annotation (MAGMA) within FUMA [33, 37]. We performed MAGMA’s gene-property test to analyze gene expression patterns across 53 tissue types from GTEx v8 [38, 39], evaluating associations between tissue-specific gene expression and genetic variants. Next, we employed the GWAS Analysis of Regulatory or Functional Information Enrichment with LD correction (GARFIELD) [40]. GARFIELD integrates GWAS findings with regulatory and functional annotations from 424 cell types and tissues, utilizing data from the ENCODE [41], and Roadmap Epigenomics projects [42]. Using a generalized linear model framework, GARFIELD calculates odds ratios (ORs) across nine GWAS P-value multiple genome-wide significance thresholds (*T* < 1 to *T* < 10⁻⁸) to quantify tissue enrichment.

For cell-type-specific enrichment, we applied stratified linkage disequilibrium score regression (S-LDSC) by integrating meta-GWAS with single-cell chromatin accessibility (scATAC-seq) and single-cell gene expression (scRNA-seq) data, for high-resolution cell-type characterization [43, 44]. Specifically, S-LDSC assessed heritability enrichment of cell-type annotations, enabling the identification of significant cell-type associations [43]. This approach accounts for the variation in brain cell composition and gene expression across developmental stages, which may help elucidate the biological mechanisms underlying the observed genetic associations. Cell-type annotations were derived from four datasets: (1) fetal brain scATAC-seq [45], (2) fetal brain scRNA-seq [46], (3) adult brain scATAC-seq [47], and (4) adult brain scRNA-seq [48]. Heritability enrichment was quantified using the standardized effect size (τ*), defined as the proportional change in per-SNP heritability associated with a one standard deviation increase in annotation value [44, 49]. Statistical significance was assessed using Bonferroni correction for τ* P-values.

Finally, genes mapped to shared loci were assessed using the web-based tool g: Profiler (https://biit.cs.ut.ee/gprofiler/gost) within Gene Ontology (GO) biological process pathways [50].

### Evaluation of gene expression associations

To investigate the expression patterns of genes mapped to shared loci, we performed transcriptome-wide association studies (TWAS) using the FUSION framework [51]. We conducted TWAS analyses across multiple tissues, including brain tissues and vitamin D-related tissues (liver, sun-exposed lower leg skin, non-sun-exposed suprapubic skin, and whole blood), utilizing GTEx v8 data [38, 39]. Genes exhibiting a pattern of genetically regulated expression, suggestive of distinct biological mechanisms or etiologies for vitD and depression, were identified by applying the Benjamini-Hochberg correction (*p*_*FDR*_ < 0.05).

### The spatiotemporal transcriptome of the human brain

To investigate developmental stage-specific gene expression patterns, we analyzed the spatiotemporal trajectories of shared genes across brain regions. Gene expression trajectories were assessed using human RNA-seq data from the PsychENCODE database (http://development.psychencode.org/*)* [52], spanning developmental stages from 8 post-conception weeks (PCW) to 40 years (PY), categorized into stages: Fetal development (8–37 PCW), Infancy (4 months-1 year), Childhood (1–13 years), Adolescence (13–19 years), and Adulthood (21–40 years). Gene expression values were quantified as reads per kilobase of transcript per million mapped reads (RPKM), subsequently log₂-transformed and mean-centered per sample. We employed non-linear LOESS regression to model temporal expression trajectories across brain tissues.

### Mendelian randomization analysis

We employed Mendelian randomization (MR) to estimate the bidirectional causal relationships between depression and vitD, implemented using the TwoSampleMR package [53]. Results were reported for inverse-variance weighted (IVW), weighted median, and MR-Egger methods [54, 55]. To mitigate potential statistical bias from sample overlap, we performed sensitivity analyses using the MRlap method [56]. This approach calculates observed MR-based effect values and then corrects them by incorporating genetic covariance estimated through LDSC.

## Results

### Genetic correlations and overlap

**Fig. 2:**
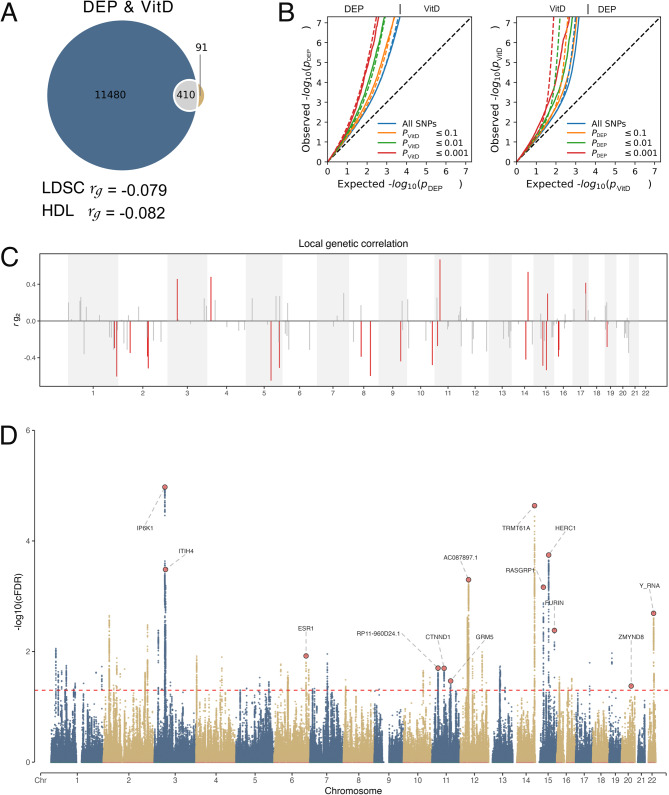
Shared Genetic Architecture Between Vitamin D Levels (vitD) and Depression (DEP). **A**. Venn Diagrams: The Venn diagram shows 410 (0.1k) shared ‘causal’ variants between vitD and DEP, estimated using MiXeR, and the genome-wide genetic correlation (rg) estimated by linkage disequilibrium score regression (LDSC) and high-definition likelihood (HDL). **B**. Conditional Q-Q Plots: Conditional Q-Q plots compare nominal and empirical -log10 *P*-values, adjusted for inflation, at significance levels of *p* ≤ 0.1, *p* ≤ 0.01, and *p* ≤ 0.001, with vitD and DEP traits being mutually conditional. All SNPs are represented by blue lines, and dashed lines indicate the null hypothesis. **C**. Shared Genetic Loci Identified through MTAG and conjFDR: Shared genetic loci jointly associated with vitD and DEP at MTAG (*p* < 5 × 10⁻⁸) and conjFDR (conjFDR < 0.05) are shown. Independent lead SNPs are highlighted in red, with the nearest genes annotated. **D**. Local Genetic Correlation: Manhattan-style plots depict regions of significant (red bars) and non-significant (gray bars) local genetic correlation between vitD and DEP

At the genome-wide level, LDSC and HDL analyses revealed a significant negative genetic correlation between depression and vitD levels (LDSC: *r*_*g*_ = −0.079, *se* = 0.017, *p* = 2.27E-06; HDL: *r*_*g*_ = −0.082, *se* = 0.021, *p* = 7.57E-05). Bivariate MiXeR demonstrated shared genetic overlap between depression and vitD beyond genetic correlation, irrespective of the direction of variant effects. Our analysis identified 11,480 (*se* = 433) trait-influencing variants for depression and 91 (*se* = 78) variants for vitD. Of these, 410 (*se* = 107) variants were shared between both traits, with 71% exhibiting discordant effects (Fig. 2A). Positive AIC values indicated an adequate model fit (Table S1 in Supplement 2). Conditional QQ plots revealed cross-trait polygenic enrichment associated with vitD and depression, showing stepwise deviations from the null hypothesis line (Fig. 2B).

At the local level, SNP-based heritability and genetic correlations were estimated using LAVA. Local SNP heritability for vitD and depression across 2,492 predefined genomic loci is presented in Figure S1 and Table S2. After the Bonferroni correction, fewer local regions remained significant. Local genetic correlation analysis, providing higher resolution of genetic overlap with mixed effect directions, revealed that 65% (18/24) of nominally significant correlations were positive between the traits (Fig. 2C; Table S3 in Supplement 2).

### Identified shared genetic loci between depression and vitamind

Through cross-trait meta-analysis by MTAG and conjFDR analysis, we identified 138 loci (MTAG *p* < 5e-8) and 74 loci (conjFDR < 0.05) shared between depression and vitD levels, respectively (Table 1). Further analysis using the FUMA pipeline refined these findings to 172 lead SNVs for depression and 78 for vitD (Figure S2 and Figure S3 in Supplement 1). Thirteen SNVs were identified by both approaches as jointly shared between depression and vitD with ten showing discordant effect directions (Fig. 2D and Table S4 in Supplement 2). Of these 13 shared SNVs, seven were located in intronic regions. These findings are broadly consistent with the genetic correlations observed. The majority of shared lead SNVs were identified in non-coding regions, with significant enrichment in intronic regions, suggesting their potential role in gene regulation. The mapped genes associated with these shared lead SNPs were linked to depression, neuropsychiatric disorders (e.g., schizophrenia, insomnia, intelligence), and several vitamin D-related traits, such as serum vitD levels, bone mineral density, and skin pigmentation [57] (Table S4 in Supplement 2).

**Table 1:** Shared genetic loci jointly associated with vitamin D (vitD) and depression (DEP) at MTAG (*p* < 5 × 10⁻⁸) and ConjFDR (conjFDR < 0.05)

CHR	LEAD_SNV	A1	A2	LEAD_BP	Mapped Genes	Func	Zscore vitD	Zscore DEP	Cor_Eff	conjFDR	MTAG pvalue
14	rs2756119	A	G	104001517	*TRMT61A*	UTR3	-5.75	6.53	No	2.30E-05	2.25E-11
11	rs708228	T	C	57585662	*CTNND1*	UTR3	3.81	-6.20	No	2.01E-02	3.36E-10
11	rs2226563	C	T	88749072	*GRM5*	intronic	-3.59	6.02	No	3.40E-02	1.09E-09
22	rs9611474	A	G	41434858	*Y_RNA*	intergenic	4.55	-5.92	No	2.03E-03	1.57E-09
15	rs62014172	C	T	63959311	*HERC1*	intronic	5.35	-5.79	No	1.80E-04	2.92E-09
20	rs4555399	A	G	45837896	*ZMYND8*	UTR3	-3.49	5.76	No	4.20E-02	5.20E-09
3	rs3774364	A	G	52863605	*ITIH4*	intronic	-5.37	5.63	No	3.28E-04	7.38E-09
15	rs56059718	A	C	38836777	*RASGRP1*	intronic	4.87	-5.59	No	6.86E-04	1.04E-08
12	rs11183747	T	C	38822595	*AC087897.1*	intergenic	-5.70	5.51	No	5.02E-04	1.34E-08
15	rs6224	T	G	91423543	*FURIN*	intronic	-4.34	5.56	No	4.14E-03	1.42E-08
11	rs7942414	C	T	28669964	*RP11-960D24.1*	intergenic	3.81	7.65	Yes	1.99E-02	1.21E-13
6	rs4870062	G	T	152237618	*ESR1*	intronic	4.00	6.77	Yes	1.20E-02	6.22E-11
3	rs7431106	A	G	49808374	*IP6K1*	intronic	-7.89	-6.44	Yes	1.06E-05	1.34E-09

The table lists the jointly associated lead SNVs in independent genomic loci shared between vitamin D and depression, identified through MTAG (*p* < 5 × 10⁻⁸) and conjFDR (conjFDR < 0.05)

*Abbreviations*: *A1* Allele 1 (effect allele), *A2* Allele 2 (noneffect allele), *LEAD_BP* Genomic position, conjFDR, conjunctional false discovery rate, MTAG, CHR, chromosome, *Cor_Eff* Concordancy of association directions between 2 traits, *Funcc* Functional category, *SNV* Single-nucleotide variant

### Functional enrichment analysis

**Fig. 3:**
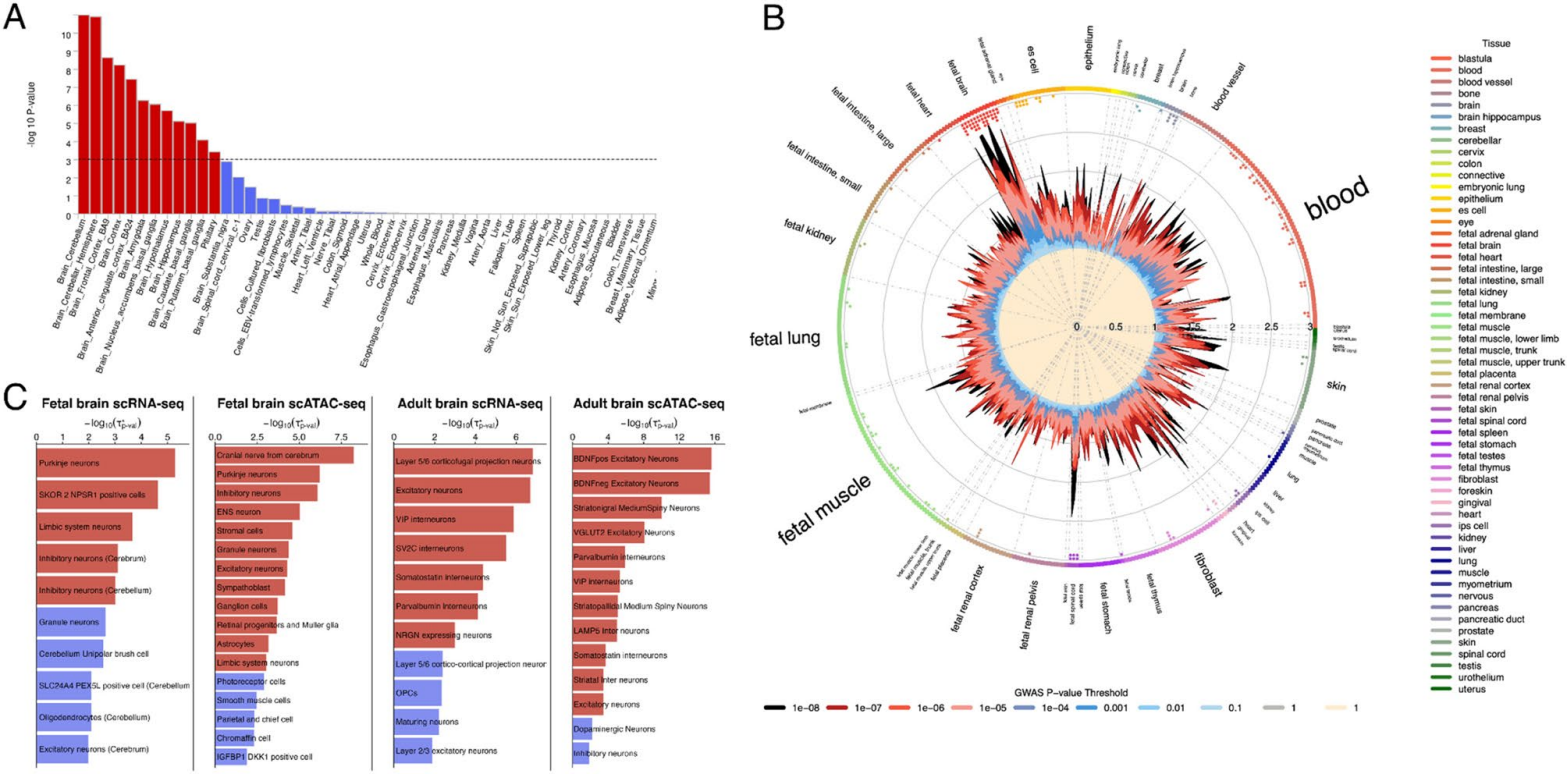
Tissue-specific and Cell type-specific Functional Enrichment for Cross-trait GWAS Meta-analysis of Vitamin D Levels and Depression. **A**. Tissue-specific Enrichment: Tissue-specific enrichment across 54 tissue types from GTEx v8 is shown as bar plots. Statistically significant results are colored red, and non-significant results are colored blue. **B**. GARFIELD Enrichment: GARFIELD enrichment is shown as wheel plots for DNase I–hypersensitive sites (hotspots). Radial lines represent odds ratios (OR) at eight GWAS *p*-value thresholds (T) for all ENCODE and Roadmap Epigenomics DHS cell lines, sorted by tissue in the outer circle. Dots in the inner ring of the outer circle indicate significant GARFIELD enrichment (if present) at *p*-value thresholds from T < 1 (outermost) to T < 10⁻⁸ (innermost), after multiple-testing correction for the number of effective annotations. Dots are colored according to the tissue or cell type tested. **C**. Cell-type-specific Enrichment: Bar plots display -log₁₀ *p*-values for positive τ∗ values in fetal and adult brain scRNA-seq and scATAC-seq cell type annotations. Statistically significant results are shown in red, while non-significant results are shown in blue

To investigate the biological mechanisms, we conducted tissue-specific and cell-type-specific enrichment analyses using multiple approaches. For tissue-specific enrichment via MAGMA, we observed that most signals were concentrated in broadly defined brain tissues, with the cerebellum showing the most significant enrichment (Fig. 3A; Table S5 in Supplement 2). Additionally, we applied GARFIELD, which uses generic regulatory annotations denoting open chromatin hotspot regions in 424 cell lines and primary cell types. GARFIELD analysis corroborated the MAGMA findings and revealed additional significant enrichment patterns in brain, cerebellar, and bone tissues, as well as in fetal brain and spinal cord tissues, suggesting developmental involvement of shared genetic foundation between depression and vitD regulation (Fig. 3B; Table S6 in Supplement 2).

Cell-type-specific enrichment analysis revealed distinct patterns between fetal and adult stages. Using scATAC-seq and scRNA-seq data from 83 fetal and adult brain cell types, we quantified the cell-type enrichment effects with τ* scores. Both scRNA-seq and scATAC-seq analyses demonstrated consistent enrichment in Purkinje neurons, limbic system neurons, and inhibitory neurons across developmental stages. However, adult-specific analyses revealed distinct enrichment patterns, predominantly in excitatory neurons, VIP interneurons, and parvalbumin interneurons (Fig. 3C; Table S7 in Supplement 2). These findings highlight stage-specific enrichment patterns, with distinct cell types involved in fetal and adult brain development through different cellular mechanisms.

Gene-set analysis of genes mapped to lead SNPs shared between depression and vitD demonstrated enrichment in Gene Ontology (GO) terms related to *molecular function regulator activity* (GO:0098772), *neuron spine* (GO:0044309), and *dendritic spine* (GO:0043197). These findings are similar to cell-type-specific enrichment patterns observed in several neuronal subtypes.

### Transcriptome-Wide association analysis

**Fig. 4:**
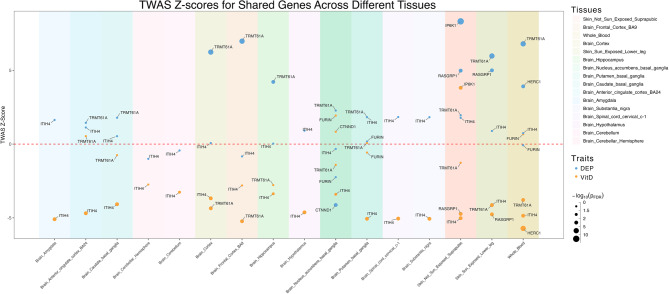
Manhattan-style Z-score plot of gene expression associated with vitamin D levels and depression in TWAS

We observed broadly opposing gene expression patterns for shared genes through TWAS across multiple brain tissues and other vitamin D-related traits (e.g., liver, skin—both sun-exposed lower leg and not sun-exposed suprapubic), as shown in Fig. 4. Of the 13 genes mapped to shared loci, six demonstrated significant associations with both vitamin D and depression after Benjamini-Hochberg correction (*P*_*FDR*_ < 0.05) in most brain tissues, whole blood, and vitamin D-relevant tissues. Notably, no genes showed significant associations with both phenotypes in liver tissue. The most frequently associated genes, *ITIH4* and *TRMT61A*, exhibited distinctly opposite gene expression patterns across most brain tissues. Although some genes were not significantly associated with vitD, this may be due to the limited statistical power of the original GWAS.

### Distinct spatiotemporal brain expression trajectories across the lifespan

**Fig. 5:**
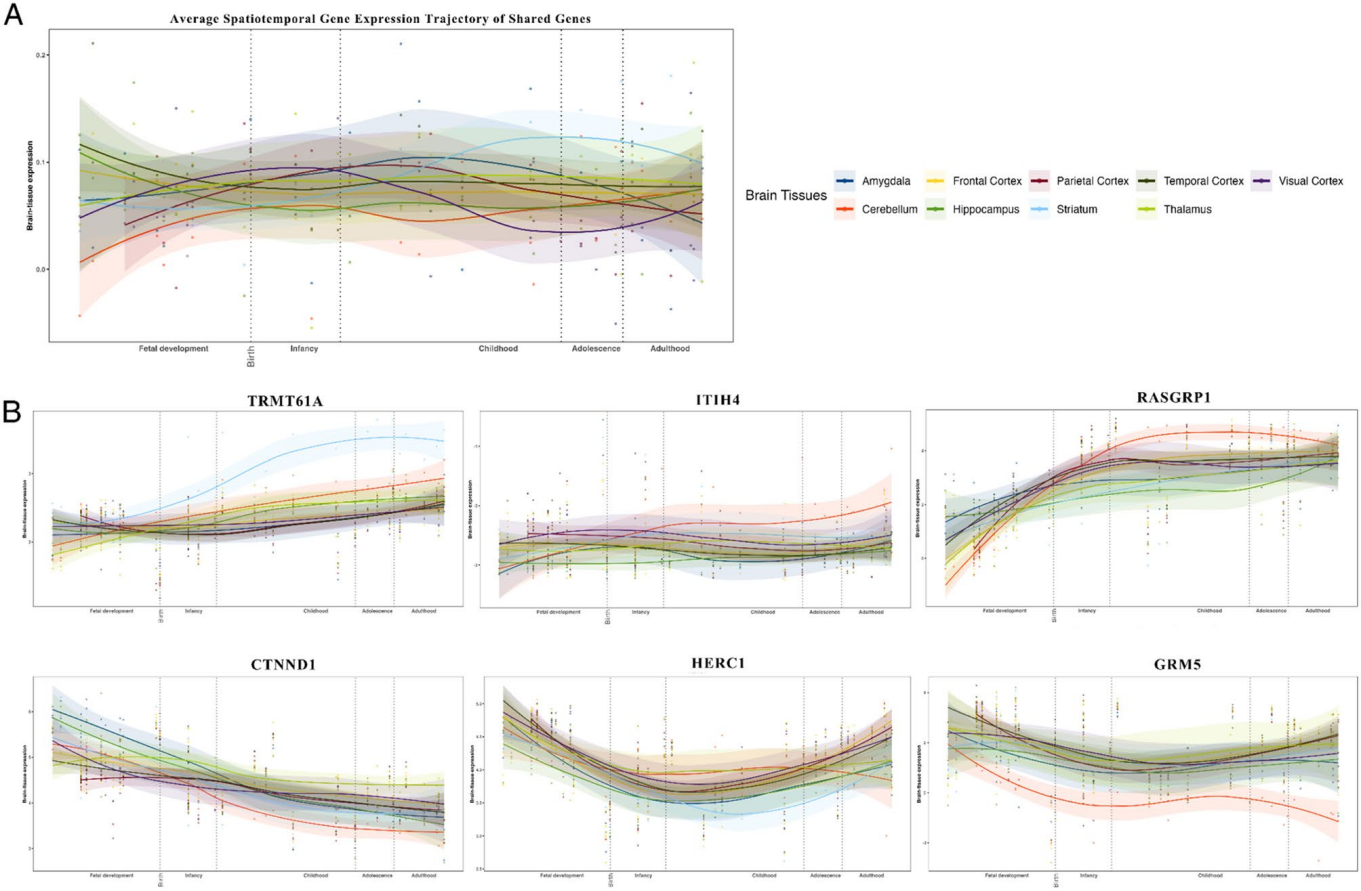
Lifespan Expression Trajectory of Shared Genes in Brain Tissues. **A**. Average Spatiotemporal Gene Expression Trajectory of Shared Genes. **B**. Spatiotemporal Gene Expression Trajectory of Each Individual Shared Gene. Brain tissue expression (y-axis) was log-transformed using the median value. Nonlinear LOESS regression lines, with 95% confidence intervals (shaded areas), were fitted to illustrate the expression trajectory for each brain tissue. The trajectory is divided into five stages: fetal development, infancy, childhood, adolescence, and adulthood

Given the critical role of coordinated gene expression in brain development and neuropsychiatric disorders [58], analysis of the transcriptional landscape of shared genes may provide insights into the neuropathology of depression. Our spatiotemporal analysis characterized the lifespan trajectories of the human brain transcriptome, extending gene expression patterns derived from TWAS results across diverse developmental stages. Ten of the thirteen shared genes were accessible in the PsychENCODE brain transcriptome, exhibiting distinct expression patterns during development and adulthood, consistent with functional enrichment analyses (Fig. 5). These ten genes displayed gradual expression trajectories across nine brain tissues from fetal development through adulthood, segregating into two distinct clusters: *(i)* Increasing Trajectory Cluster: Characterized by progressively increasing expression from development to adulthood, including *TRMT61A*, *ITIH4*, *RASGRP1*, *IP6K1*, *FURIN*, and *ESR1*. *(ii)* Decreasing Trajectory Cluster: Marked by gradual decline in expression across brain tissues, comprising *CTNND1*, *HERC1*, *GRM5*, and *ZMYND8* (Fig. 5B; Figure S4 in Supplement 1). The distinct clustering of gene expression trajectories over the lifespan aligns with cell-type-specific functional enrichment results, supporting the neurodevelopmental hypothesis of depression pathology.

### Absence of causal association between depression and vitamin D levels

Our bidirectional MR results revealed no significant genetic causal relationship between vitD and depression, as assessed using multiple MR methods (MR-Egger, weighted median, inverse variance weighted [IVW], simple mode, and weighted mode) implemented in the TwoSampleMR package (Figure S5 in Supplement 1; Table S9 in Supplement 2). The primary IVW analysis showed no significant effect of vitD exposure on depression (*β* = −0.025, *se* = 0.016, *p* = 0.121) or depression exposure on vitD levels (*β* = −0.003, *se* = 0.026, *p* = 0.913). Sensitivity analysis using MR-Lap, which corrects for potential sample overlap bias, corroborated these findings, demonstrating non-significant associations for vitD exposure on depression (corrected *β* = −0.005, *se* = 0.011, *p* = 0.672) and depression exposure on vitD (corrected *β* = −0.084, *se* = 0.041, *p* = 0.039). After Bonferroni correction for multiple testing, no significant corrected effect estimates were observed in either direction, corroborating the Two-Sample MR results.

## Discussion

In this study, We conducted comprehensive genetic analyses to investigate the relationship between vitD levels and depression. Our study extended beyond genetic correlation analyses to quantify trait-specific and shared genetic variants. Through cross-trait GWAS meta-analysis and conjFDR analyses, we identified 13 genetic loci shared between these traits. Functional enrichment analyses of shared genetic signals, conducted at both tissue and cell-type levels, revealed significant involvement in brain development processes. Genes mapped to the shared loci exhibited distinct expression patterns and lifespan expression trajectories, further supporting the functional enrichment findings. Our MR results showed no evidence of a genetically causal effect of vitD on depression or vice versa. These results suggest potential biological mechanisms through which vitD regulation may influence depression risk, particularly via neurodevelopmental pathways.

Previous epidemiological and clinical studies, including both cross-sectional and cohort designs, have consistently demonstrated an inverse association between plasma vitD concentrations and depression, including both symptomatic presentation and major depressive disorder [59–61]. Our genetic findings align with these observations, revealing negative genetic correlations at both genome-wide and regional levels. Moreover, Bivariate MiXeR analysis provided evidence for shared genetic architecture, with 76% of identified variants showing discordant effects. The observed distribution of directional effects among shared loci supports epidemiological findings suggesting a co-occurrence of low vitD levels with depression. Cross-trait GWAS meta-analysis confirmed these pleiotropic effects, with conjFDR analysis identifying 13 shared genetic loci, suggesting common biological pathways underlying both conditions.

The relationship between VDD in pregnant women and an increased risk of depression in their offspring highlighting the vital role of vitD in brain development. Our functional enrichment analyses revealed shared genetic signals between vitD levels and depression specifically in fetal brain tissue. Developmental VDD has been reported associated with altered brain morphology, including decreased cortical thickness and enlarged lateral ventricles [62]. A birth cohort study found that offspring exposed to deficient vitD levels during gestation have an increased risk of developing depression during adolescence, compared to those exposed to normal vitD levels [63]. Additionally, developmental VDD deficient neonates show decreased levels of neurotrophins (NGF and GDNF) and p75NTR receptor expression, with these deficits persisting into adulthood [64]. Cell type-specific enrichment analyses revealed distinct developmental patterns. Fetal brain scRNA-seq and scATAC-seq sequencing data demonstrated significant enrichment in Purkinje neurons, limbic system neurons, and inhibitory neurons. In contrast, adult brain analyses showed enrichment predominantly in excitatory neurons, VIP interneurons, and parvalbumin interneurons. Evidence suggests that both excitatory glutamate neurons and inhibitory GABA interneurons mediate neurobiological changes in cortical and limbic regions, contributing to network dysfunction in depression [65, 66]. Purkinje neurons, which express diverse excitatory amino acid transporters (EAATs), modulate glutamate transporter expression and metabotropic glutamate receptor (mGluR1) stimulation, exhibiting multiple forms of synaptic plasticity [67]. Vitamin D plays a crucial role in maintaining calcium homeostasis within Purkinje cells, modulating their developmental maturation and supporting the integrity of cerebellar circuitry [68]. Inhibitory neurons, primarily GABAergic, follow a prolonged developmental timeline and their dysfunction—manifested through genetic mutations in cortical interneurons, altered neuronal density, or reduced GABA neurotransmitter levels—has been implicated in depression pathophysiology [69]. Vitamin D has also been shown to reduce the expression of GABA transporter 3 (GAT3), essential for GABA reuptake, and excitatory amino acid transporters (EAATs). These findings suggest that vitamin D influences both inhibitory and excitatory neurotransmission through the regulation of neurotransmitter transport systems [70]. Recent research by Kasatkina et al. demonstrated that VDD induces presynaptic dysfunction and proinflammatory responses as early events in disease pathogenesis, providing evidence for vitamin D’s neuroprotective role [71]. Integrative analyses of single-cell transcriptomics and Allen Human Brain Atlas expression datasets have identified specific neuronal populations—comprising somatostatin interneurons, VIP interneurons, parvalbumin interneurons, and astrocytes—as consistent cellular correlates of depression, corroborating our adult brain cell type-specific enrichment findings [72].

Using TWAS analysis, we assessed the expression of shared genes and found that their expression levels exhibited distinctly opposite patterns across most brain tissues and vitamin D-related traits. These shared genes demonstrate involvement in critical neurobiological pathways, including neuroimmunomodulation, neurotrophic factor regulation, neuroprotection, neuroplasticity, and neurodevelopment. Gene Ontology enrichment analysis indicated that these shared genes are involved in molecular function regulator activity and dendritic spine development and maintenance. Specifically, *ITIH4*, a member of the inter-alpha-trypsin inhibitor family of serine protease inhibitors, has been reported to be associated with other neuropsychiatric conditions, including schizophrenia and Alzheimer’s disease [73–75]. A proteomic analysis of peripheral serum from patients with major depressive disorder revealed elevated ITIH4 levels, consistent with our TWAS findings of broadly increased *ITIH4* gene expression across brain tissues [76]. *TRMT61A*, a primary enzyme responsible for N1-methyladenosine (m1A) modification in mitochondria, catalyzes a reversible modification targeting rRNAs and tRNAs. The m1A modification enhances tRNA structural stability and promotes proper tRNA folding, processes that have been implicated in Alzheimer’s disease pathogenesis [77, 78]. *CTNND1*, which encodes catenin (cadherin-associated protein), has been reported to play a crucial role in regulating dendritic spines and synapse development [79]. Through GWAS meta-analysis, Luo et al. identified *CTNND1* as a risk gene associated with depression and anxiety [80, 81]. Inositol hexakisphosphate kinase 1 (*IP6K1*) regulates neuronal migration and vesicular glutamate transport, processes implicated in the pathophysiology of depression [82, 83]. Estrogen receptor alpha (*ESR1*) regulates gene expression and estrogen signaling in brain regions implicated in depression [84]. Furthermore, *ESR1* modulates multiple molecular pathways through its interaction with vitD, including immune and inflammatory responses, neurotransmitter activity, endothelial and vascular processes, and hormonal alterations [85].

The MR analyses provide no evidence for bidirectional causal effects between vitD and depression consistent with previous MR studies [86, 87]. While recent meta-analyses of randomized controlled trials of vitD supplementation have found evidence for reductions in depression and depressive symptoms [88, 89]. However, The presence of a threshold effect has been proposed, where disease risk and benefits of vitD supplementation may only surface below certain thresholds of vitD status which can not be found using the standard linear MR approach. This may explain the null findings from MR studies assessing the effect of vitamin D status on depression risk. Recently, Bassett et al. observed a suggestive association with probable lifetime major depression in the stratum of the population with the lowest levels of vitD using non-linear MR analyses [90]. This suggests that vitD supplementation may reduce risk of depression for those with low vitD levels. These findings suggested that vitD supplementation might be beneficial in reducing depression risk specifically for individuals with vitamin D deficiency.

Several limitations should be considered when interpreting these findings of this study. Firstly, our analyses were restricted to individuals of European ancestry, potentially limiting the generalizability of results to other ancestral populations. Further studies in diverse ethnic groups are recommended. Second, although we employed robust genetic statistical approaches—including LDSC, HDL, LAVA, MTAG, and conjFDR, which are designed to mitigate potential bias from sample overlap, we cannot completely exclude the possibility that overlapping participants may have inflated the cross-trait enrichment results. Moreover, given that both depression and vitamin D levels are polygenic and complex traits, they are inherently influenced by various confounders (e.g., environmental factors), which could potentially bias pleiotropy analyses. Lastly, our investigation into the shared genetic content of identified variants relied primarily on silico methods. Additionally, while our results suggest involvement of brain development pathways, they may also reflect more general associations with brain-expressed genes. Laboratory-based experimental validation of these mechanisms is warranted.

## Conclusion

Our study revealed shared genetic content between depression and vitD levels, identifying 13 shared genetic loci. And, tissue-specific and cell-type-specific functional enrichment analyses illuminated pathogenic neurodevelopmental processes underlying depression. The mapped genes at these shared loci, supported by previous findings, suggest that vitamin D-related mechanisms may modulate resilience to adverse environmental factors during neurodevelopment. These findings provide potential directions for future investigation through animal models and longitudinal studies in large birth cohorts.

## Supplementary Information

Additional File 1: Supplementary Figures.

Additional File 2: Supplementary Tables.

## Data Availability

No datasets were generated or analysed during the current study.

## Abbreviations

YLDs: Years lived with disability
vitD: Vitamin D
UKBB: UK Biobank
VDD: Vitamin D deficiency
GWAS: Genome-wide association studies
LDSC: Linkage disequilibrium score regression
MiXeR: Bivariate causal mixture model
MTAG: Multi-Trait Analysis of GWAS
conjFDR: Conjunctional false discovery rate analysis
TWAS: Transcriptome-wide association studies
AIC: Akaike Information Criterion
Q-Q: Conditional Quantile–Quantile plots
HDL: High-definition likelihood
LD: Linkage disequilibrium
LAVA: Local analysis of co-variant annotation
SNVs: Single-nucleotide variants
CADD: Combined Annotation Dependent Depletion scores
MAGMA: Multi-marker Analysis of Genomic Annotation
GARFIELD: GWAS Analysis of Regulatory or Functional Information Enrichment with LD correction
S-LDSC: Stratified linkage disequilibrium score regression
scATAC-seq: Single-cell chromatin accessibility
scRNA-seq: Single-cell gene expression data
GO: Gene Ontology
MR: Mendelian randomization
IVW: Inverse-variance weighted
