# Supplementary material for: The relationship between vitamin D levels and depression: a genetically informed study
Source: Nutr J. 2025 Oct 10;24:158. doi: 10.1186/s12937-025-01199-1 (PMC12512799; doi:10.1186/s12937-025-01199-1)
Supplement: Supplementary file 1 — Additional File 1: Supplementary Figures. [file 12937_2025_1199_MOESM1_ESM.docx]

**The Relationship Between Vitamin D Levels and Depression: A Genetically Informed Study**

Honggang Lyu **^1,#^**, Lijun Kang**^1,#^**, Qian Gong **^1^**, Xin-Hui Xie **^1^**, Simeng Ma **^1^**, Lihua Yao**^1^**, Mian-mian Chen **^1^**, Lingfeng Zhang **^1^**, Hao Yu **^2^**, Xubo Wang **^3^**, Chao Wang **^1,^**^*^, and Zhongchun Liu **^1,4,^**^*^

**Supplementary Figures**

Figure S1. Local SNP heritability.

Figure S2: Results of Cross-Trait GWAS Meta-Analysis Using MTAG

Figure S3: Conjunctional FDR Manhattan Plot.

Figure S4. Lifespan Expression Trajectory of Shared Genes in 9 Brain Tissues.

Figure S5: Scatter Plot of Bidirectional Genetic Causal Relationships Between Vitamin D Levels and Depression

**Supplementary Figures**


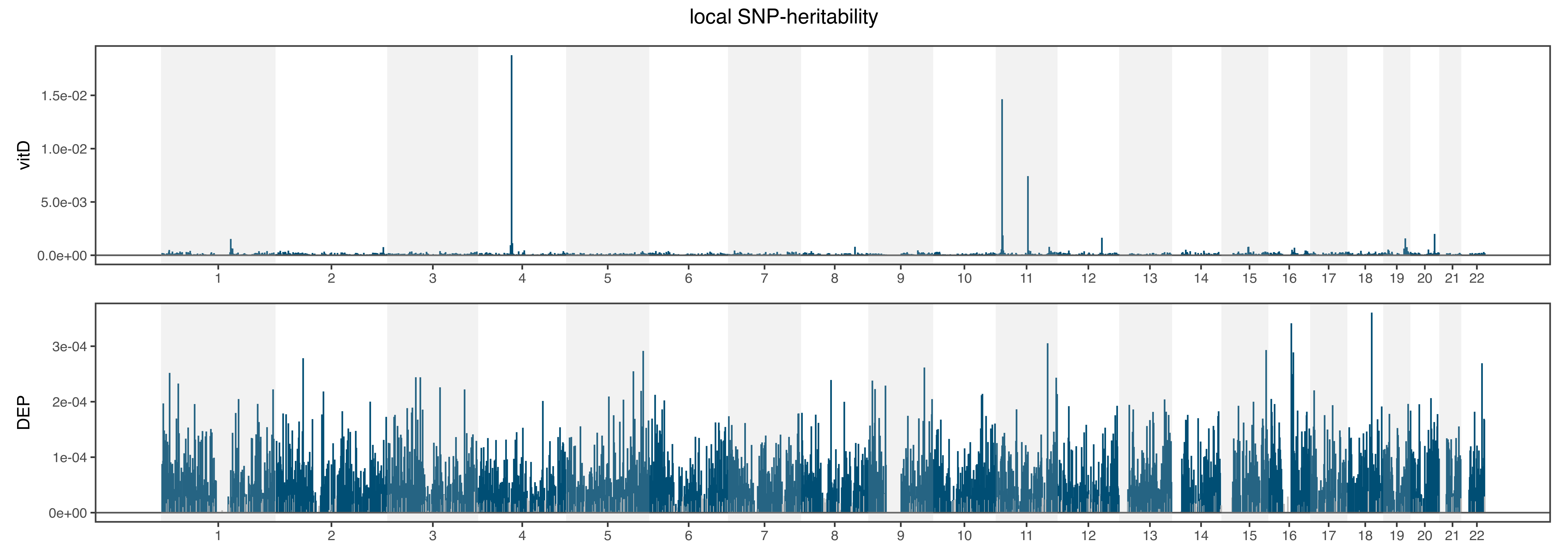


**Figure S1. Local SNP heritability.**

Manhattan-style plots display local SNP heritability for vitmamin D (vitD) levels and depression (DEP). Significant local SNP heritability across the predefined 2,495 regions is indicated by blue bars, while non-significant regions are shown in gray.


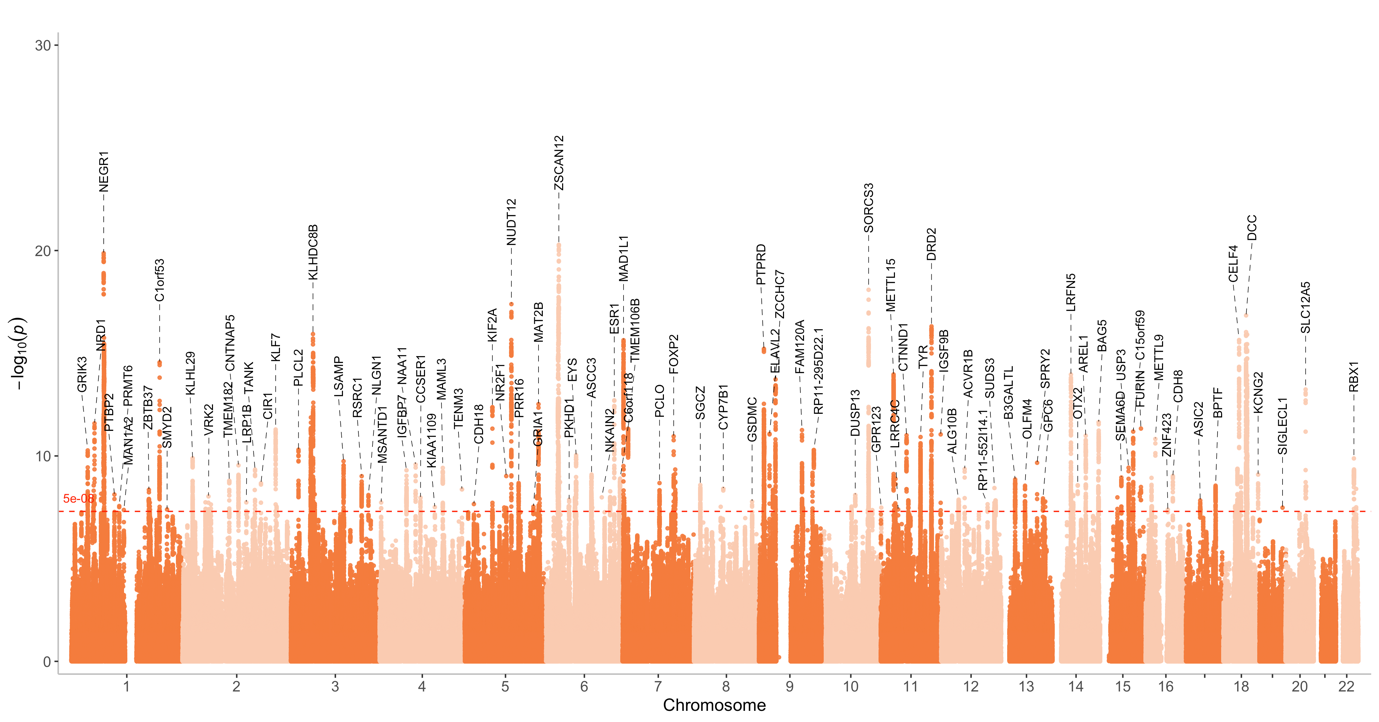


**Figure S2: Results of Cross-Trait GWAS Meta-Analysis Using MTAG**

The figure illustrates the results of a cross-trait GWAS meta-analysis performed with the Multi-Trait Analysis of GWAS(MTAG) method. A total of 172 Lead SNVs with MTAG *P* < 5e-8 are annotated with their nearest genes.


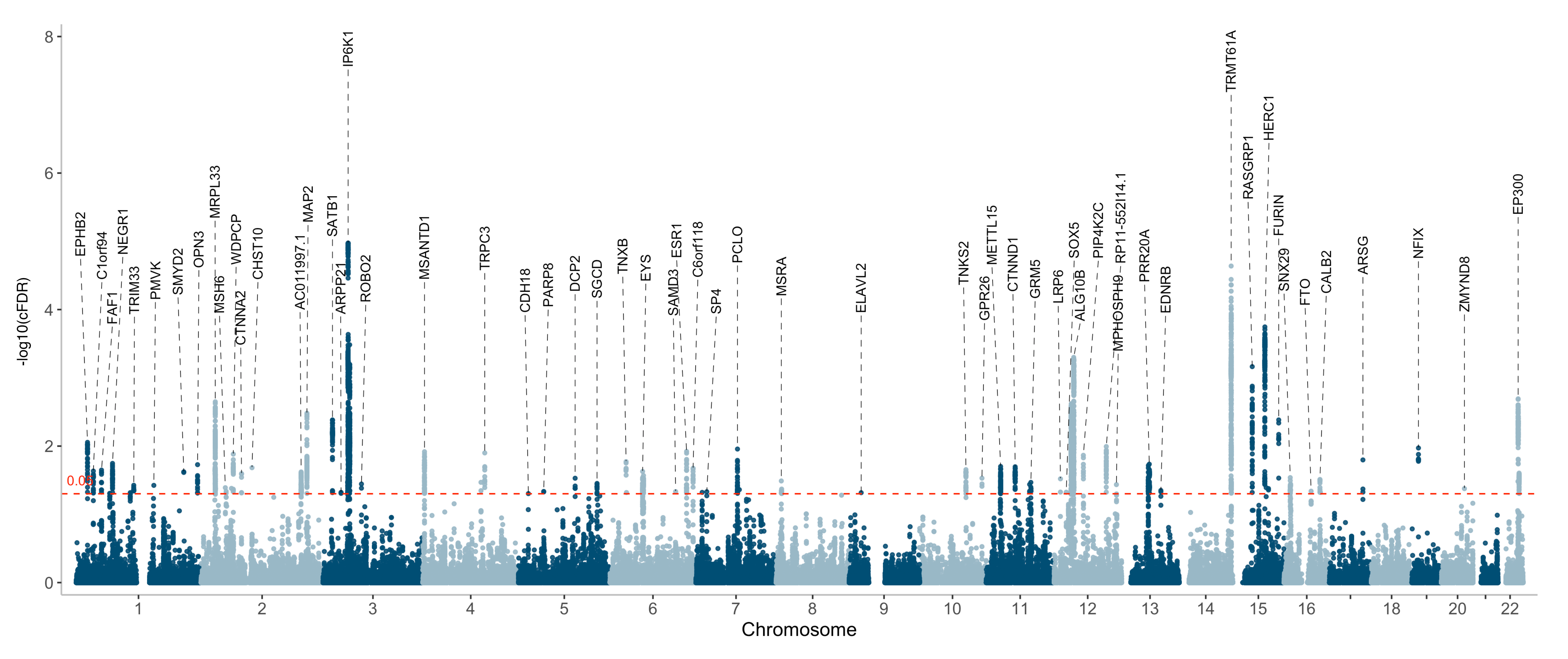


**Figure S3: Conjunctional FDR Manhattan Plot.**

The figure presents a conjunctional false discovery rate (conjFDR) Manhattan plot. A total of 78 lead SNVs with conjFDR < 0.05 are annotated with their nearest genes.


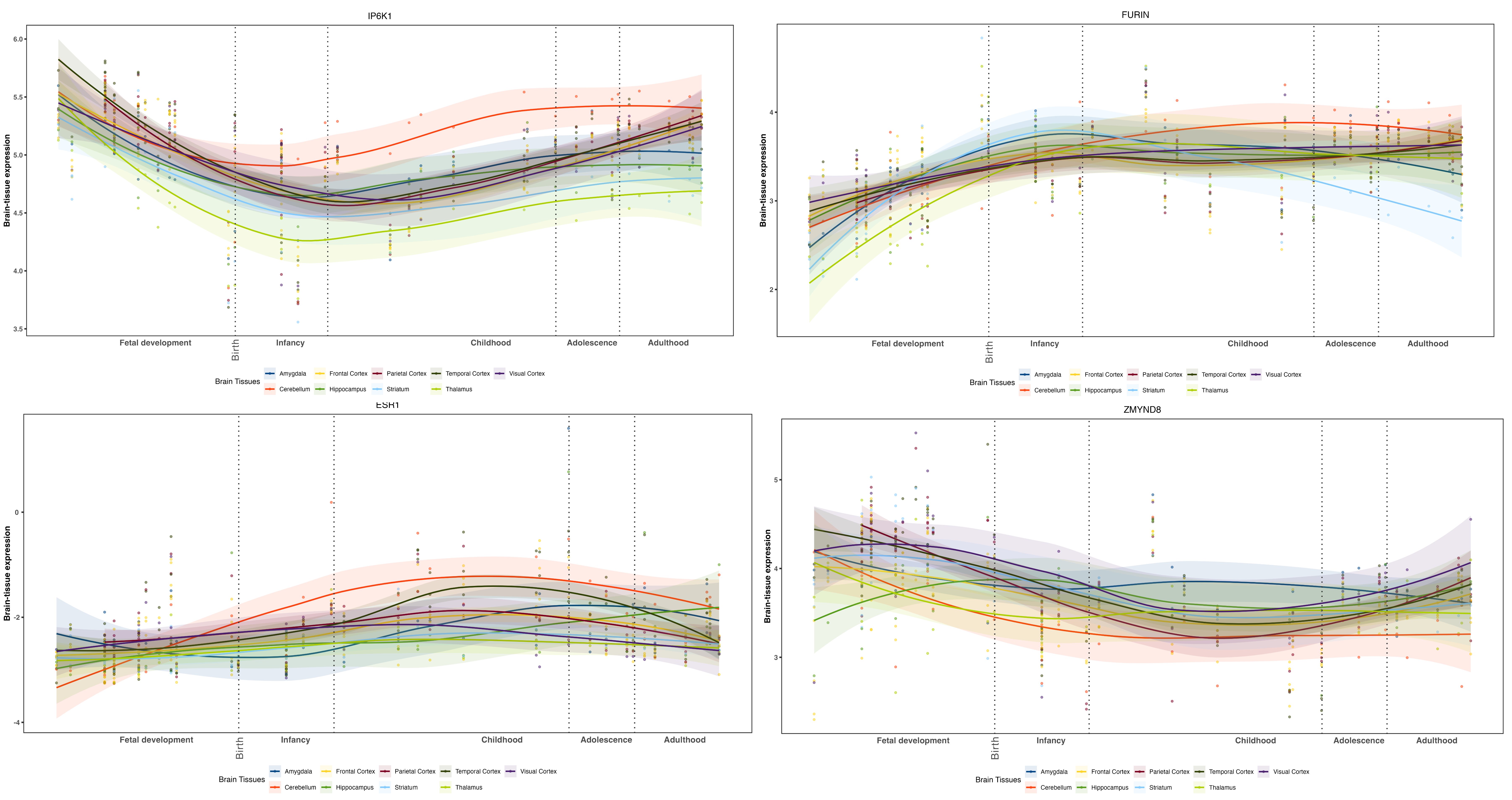


**Figure S4. Lifespan Expression Trajectory of Shared Genes in 9 Brain Tissues.** Spatiotemporal Gene Expression Trajectory of Each Individual Shared Gene. Brain tissue expression (y-axis) was log-transformed using the median value. Nonlinear LOESS regression lines, with 95% confidence intervals (shaded areas), were fitted to illustrate the expression trajectory for each brain tissue. The trajectory is divided into five stages: fetal development (8 PCW ≤ age < 38 PCW), infancy (38 PCW ≤ age < 1 year), childhood (1 year ≤ age < 12 years), adolescence (12 years ≤ age < 20 years), and adulthood (20 years ≤ age ≤ 60 years).

**
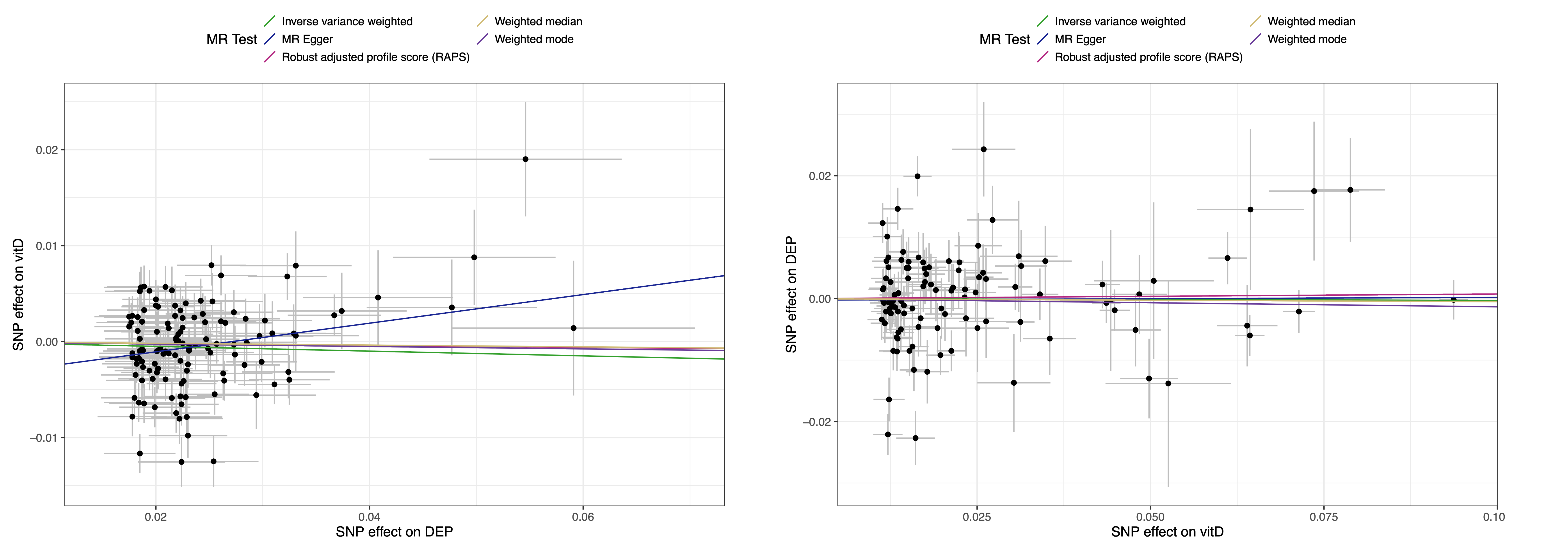
**

**Figure S5: Scatter Plot of Bidirectional Genetic Causal Relationships Between Vitamin D Levels and Depression**
